# The Guidelines for Application of Kinesiology Tape for Prevention and Treatment of Sports Injuries

**DOI:** 10.3390/healthcare8020144

**Published:** 2020-05-26

**Authors:** Adéla Andrýsková, Jung-Hoon Lee

**Affiliations:** 1 Integrated Physical Medicine Institute, Dong-Eui University, Busan 47340, Korea; andryska8@gmail.com; 2 Department of Physical Therapy, College of Nursing, Healthcare Sciences and Human Ecology, Dong-Eui University, Busan 47340, Korea

**Keywords:** kinesiology taping, tape’s application, sports taping, rules of taping, prevention, side-effects, core’s stability

## Abstract

The number of kinesiology tape’s users is increasing year by year. However, the insufficiency of fundamental knowledge about the appropriate usage of kinesiology tapes can generate undesired side-effects caused by incorrect application of kinesiology tapes and/or denouncement of kinesiology tapes as an ineffective practice. Therefore, it is necessary to arrange a set of general guidelines of kinesiology taping that must be followed. If not, the treatment may have to be ceased due to the side-effects such as skin’s troubles. Another problem, which impeaches effectivity of treatment by kinesiology tapes, is focusing solely on the present area of pain or discomfort. However, such solution is only short-termed and the likelihood of reappearance of the pain is remarkably high. Therefore, it is essential to find and eliminate the origin of the problem. If these fundamentals conditions of tape’s application are satisfied, the treatment by kinesiology tapes may bring us far more better results.

The popularity of kinesiology taping across the world is rising. This fact is supported by the common appearance of kinesiology tape on athlete’s bodies during top events such as the Olympic games or football leagues over the last decade. Therefore, the results of the application of kinesiology tape will be important to sports physiotherapists, trainers, and sports medicine physicians.

Even though kinesiology tape was originally developed and used for the treatment of injuries, joint stabilization, and pain reduction, nowadays, it is particularly valued for its injury prevention and performance enhancement properties [1]. Currently, the public attention of kinesiology tape is nothing surprising if we consider all the advantages the application of this elastic cotton fabric tape can provide [2]. Kinesiology taping, in comparison to many other treatments, is invasive, simple, affordable, does not cause pain, and a relatively small amount of time is required for the application [3]. However, recently, there have been arguments and studies that object against its usage and denounce its effectiveness.

It is important to remember that several techniques of kinesiology taping exist and the application of kinesiology tape for certain pain, injury prevention, performance enhancement, etc., can differ significantly depending on each technique. Nevertheless, a basic set of rules for the application of kinesiology tape to prevent any undesired side-effects should be strictly followed when using any techniques.

The most fundamental rule is that the skin of the taping subject must be clean, without any dirt, oils, or sweat [3]. Also, longer body hairs, which could hinder proper adhesion of the tape to the skin, must be shaved prior to taping [3]. However, besides these well-known principles, there is another particularly important and fundamental rule of kinesiology taping that is either unknown or not respected, application of tape for a maximum of 24 hours [3]. Subjects should never wear kinesiology tapes for more than a day [3]. There are several reasons for this time limitation. Skin troubles are the most frequent problems that arise due to excessive wearing of kinesiology tape [3]. This does not solely mean allergic reactions that can be provoked by the glue, which adheres the fabric to the skin. Skin problems may arise particularly due to perspiration, which is part of everyday activities. If kinesiology tape contaminated by sweat is worn for more than one day, it may cause skin irritation [3]. It is also recommended to remove the tape immediately after showering as the wet tape can provoke undesirable skin effects. If undesirable side-effects (as itchiness, skin irritation, etc.) appear, the successive application of kinesiology tape is impossible, and treatment must be ceased [3]. Thus, increased attention needs to be paid to the condition of the skin. If itchiness or another undesirable effect appears, kinesiology tape must be removed immediately [4,5,6]. 

The 24 hours application rule is also recommended due to the constantly changing physical condition of a subject. Where kinesiology tape is applied, and the level of stretching of the tape may vary based on treatment progression. Therefore, taping techniques need to be modified day by day as the treatment continues [4,5,6]. 

Another fundamental factor is the skin’s adaptation to stimulation created by kinesiology taping [6]. In order to provide effective skin stimulation, it is necessary to apply new kinesiology tape every day [7,8,9]. 

Not stretching the origin and insertion areas of kinesiology tape is one of the basic rules of taping as well. Approximately 2–3 cm of the starting and ending point of the tape needs to be applied to the skin without any stretching [6]. Violation of this rule may cause skin problems and cause discontinuation of the treatment [4,10,11]. 

Even though one of the biggest advantages of kinesiology taping is the elasticity of the tape, it is not recommended to stretch the tape excessively. This principle also applies to skin stretching. The skin should not be stretched excessively before the application of tape. After the application of tape, it is necessary to assure that the tape adheres to the skin properly, but vigorous rubbing of the tape should be avoided. These recommendations are made to prevent skin irritation [3]. 

In order to prevent skin problems in some body parts (e.g., anterior aspect of the acromion), it is recommended to apply hypoallergenic undertape (50 × 75 mm) to the origin and insertion areas before applying kinesiology tape [3,4]. 

As for the kinesiology taping of athletes, it is essential to give at least 10 min for the body to adapt to the taping before any activity. If excessive perspiration during physical activity occurs, athletes should take a shower afterward and take off the kinesiology tapes when still wet.

It is not recommended to apply kinesiology tapes to the abdominal area immediately after food consumption. Otherwise, mild digestive troubles can be generated [3].

These rules are especially important for the prevention of any skin irritation or discomfort that can occur by wearing the tapes. However, there is another fundamental principle of kinesiology taping (and treatment overall) that tends to be overlooked. Many practitioners focus only on the part of body that needs to be fixed, but it is essential to see the body as a whole system. Segments of the human body are interconnected by the kinetic chain [12]. Therefore, a movement of one body part influences another one. In the case of athletes, physicians tend to concentrate on limbs, as those are perceived as a base for movement. However, since the core muscles serve as a stable basement for limbs’ movement, it is necessary to stabilize the core muscles first to achieve the improvement of limb movement [13]. In other words, to improve the movement of distal body parts, the proximal parts must be stabilized first [14]. 

In a recently published study [15], kinesiology tape was applied to the forearm flexor muscles used for sport climbing, but there was no immediate effect on sport climbing performance and muscular strength. As sport climbing involves complex and several moves, they should be performed with caution [16]. Sport climbing can be considered an extreme sport where a climber often repeats moves, twisting or stretching the upper and lower limbs to grab or step on a distant hold by hand or foot. The sport also involves the use of postural muscles responsible for balance and coordination [16]. The transverse abdominis muscle, which increases abdominal pressure, contracts before a movement in the upper limb segment [17,18], increasing the stiffness of the lumbar spine [19]. In contrast, the contraction of the oblique abdominal muscles provides stability regardless of the direction of limb movement [20]. Hence, the results of the Limmer et al. [15] study may have been different if the tapes had been applied not just to the core muscles, which provide stability to the trunk but also the shoulder muscles that connect the trunk and upper extremities, finger flexor, and lumbrical muscles. 

The same principle applies even for chronic pain in limbs. In such a case, the physician should not concentrate only on the elevation of the pain but should find the origin of the pain, which may be located in proximal parts of the body. Otherwise, taping may be ineffective, or the pain will be relieved only temporally and reappear [3].

Many other factors need to be taken into consideration while choosing the appropriate taping technique to achieve desired results, but the principles described above should never be omitted. If a solid foundation of rules for taping is established and current techniques are enhanced (or substituted by new ones), treatment by kinesiology tape may bring more various and effective results than is expected.
